# National Health Insurance unpacked: Part 3: Registration of patients at primary care facilities

**DOI:** 10.4102/safp.v62i1.5214

**Published:** 2020-09-09

**Authors:** Bob Mash

**Affiliations:** 1 Division of Family Medicine and Primary Care, Faculty of Medicine and Health Sciences, University of Stellenbosch, Cape Town, South Africa

## Introduction

This four-part series in the *South African Family Practice* journal unpacks the details of national health insurance (NHI) as proposed in the NHI Bill that was submitted to Parliament in July 2019.^1^

In Part 3 of the series we look at the registration of patients for NHI.

## How will registration of patients be organised?

All those covered by NHI will need to register with the Fund.^2^ All South African citizens, permanent residents, children (regardless of origin), refugees and prison inmates will be eligible for all benefits. Asylum seekers and illegal migrants will be eligible for treatment of emergencies and notifiable diseases.

Parents will need to register their children and any adolescent between the ages of 12 and 18 years, who is not a dependent, can also apply for registration. A supervising adult must apply for children from child-headed households to be registered and if necessary any employee of the accredited provider or establishment can assist such a child to be registered. Going forward, a child born to a registered user will automatically be registered at birth.

Everyone that is registered will be expected to access the health system via and accredited care provider or establishment.^2^ Registration is with the Fund and not with a specific primary care provider or establishment. Even if you retain additional medical insurance you will still be expected to register for NHI. Registered users will be entitled to healthcare services that are free at the point of care. The primary care provider then acts as the gatekeeper to the rest of the system and patients will be required to adhere to the referral pathways.

## Will facilities/general practitioners (GPs) be allowed to retain their patients?

Each person will have the right to utilise their preferred primary care provider or health establishment. Facilities will be able to motivate and enable their patients to utilise their services, but will not be able to negate people’s choice. Private general practices would therefore be able to retain their patients if the practice is accredited by the fund and their patients choose to utilise them.

## Is there a certain minimum number of patients needed?

The Bill does not define a minimum number of registered patients per provider. The Contracting Unit for Primary healthcare (CUP) will contract services for the catchment area of a district hospital and its finances will be primarily based on the size of the population in that area. This means that if the catchment area is over serviced, with too many accredited providers, there will be less funding available per provider. Part 4 of the series will look at remuneration. This also implies that people will need to utilise a primary care provider within the area covered by their CUP as the money for their benefits is allocated to the CUP where they live.

One should assume that the underlying capitation fee will support a sufficient number of health professionals and resources to provide the required services to the registered population for that CUP.

## What happens if patients come who are not registered?

If you are not registered then you are not covered by NHI. The intention is that people registered with the Fund will access care via a primary care provider or establishment in the area covered by the CUP, where they live. They will not, however, be obligated to use a specific primary care provider or establishment, although they must enter the system via primary care under normal circumstances. This also means that if you are away from home, on work or for holiday, you will be able to access care at any accredited primary care establishment. Proof of registration should be available on the Health Patient Registration System, which will need to be installed in every accredited establishment. The National Department of Health has already installed hardware and software for this purpose in most public sector primary care facilities. Foreigners, who are here for work or holiday, will be expected to have their own travel insurance policy to cover healthcare.

## What information about patients is needed when they register?

A South African identity document, original birth certificate or refugee identity card can be used to register. At present, the Bill says that biometrics will also be collected from people who register. It seems clear that fingerprint readers will be needed, but photographs and proof of residence are less certain. National Health Insurance will need to provide software and a fingerprint reader, but existing hardware may otherwise be sufficient.

Primary care providers or health establishments will require an information platform that includes registration of patients and will need to submit this information to the Fund’s Health Patient Registration System. All registered users will have a unique personal identity. Initial implementation of NHI must include this Health Patient Registration System and 44 million people are already on the system.

## How will patients be divided between public and private facilities?

The fund will not divide patients between public and private facilities. Patients will be able to utilise any accredited primary care provider or health establishment of their choice.
